# Effects of gadolinium on cardiac mechanosensitivity in whole isolated swine hearts

**DOI:** 10.1038/s41598-018-28743-w

**Published:** 2018-07-12

**Authors:** Hanyu Zhang, Gregory P. Walcott, Jack M. Rogers

**Affiliations:** 1 University of Alabama at Birmingham, Department of Biomedical Engineering, Birmingham, 35294 United States of America; 2 University of Alabama at Birmingham, Division of Cardiovascular Disease, Department of Medicine, Birmingham, 35294 United States of America

## Abstract

Mechanical stimulation can elicit electrical activation of the heart. This mechanosensitivity can start life-threatening arrhythmias (*commotio cordis*) or terminate them (precordial thump). Mechanosensitivity may also be involved in arrhythmogenesis in other settings. Stretch-activated ion channels (SACs) are thought to be important in mechanosensitivity and a number of agents that block them have been identified. Such agents could potentially be used as tools in experimental investigation of mechanosensitivity. However, studies using them in intact-heart preparations have yielded inconsistent results. In the present study, we used isolated, perfused hearts from 25–35 kg pigs and a computer-controlled device that repeatably delivered focal mechanical stimuli. The concentration-dependent ability of the SAC blocker gadolinium to suppress mechanical activation was assessed by the success rate of mechanical stimulation and by the delay between successful mechanical stimulation and electrical activation. In six hearts, perfusate was recirculated. In an additional six hearts, perfusate was not recirculated to prevent gadolinium from forming complexes with metabolic waste and possibly precipitating. Gadolinium did not suppress mechanically-induced activation. Although gadolinium has been shown to be an effective SAC blocker in isolated cells, using it to probe the role of mechanical stimulation in whole heart preparations should be done with great caution.

## Introduction

Mechanical function and electrical activity are bidirectionally coupled in the heart^1^. Cardiac electrical activity can be modulated by mechanical stimulation, which is known as cardiac mechanosensitivity. Generally, cardiac mechanosensitivity includes change of heart rhythm^2^ or initiation of ectopic electrical excitation in response to mechanical stimulation^3^. Both phenomena have been documented for decades.

Mechanosensitivity is an intrinsic property of the heart and has been documented in many heart preparations, including isolated heart^4,5^, isolated cardiac tissue^6^, and isolated cardiac cells^7^. Mechanosensitive ion channels (MSCs) have been thought to play a key role in cardiac mechanosensitivity. MSCs were first discovered by Guharay and Sachs in chick skeletal muscle cells in 1984^8^. Since then, several MSCs have been identified from a wide range of cells, including cardiac cells (see the review by Reed *et al*.^9^).

Stretch-activated ion channels (SACs) are a subcategory of MSCs that switch from closed state to open state in response to mechanical stretch. Among SACs, non-selective cation SACs (SAC_NS_) have been viewed as important contributors to mechanically induced electrical excitation. Those SACs conduct Na^+^, K^+^ and Ca^2+^ currents and have a linear voltage-current relationship with reversal potential ranging from −30 mV to 0 mV^10,11^. At resting potential, when activated by mechanical stretch, they depolarize the membrane potential by carrying inward Na^+^/Ca^2+^ currents and thus may trigger ectopic electrical excitation^12^. Potassium-selective SACs (SAC_K_) also have been found in cardiac cells. These channels generally have a larger conductance than that of SAC_NS_^13,14^. Being selective to potassium and having reversal potential about −70 mV, SAC_K_ channels tend to cause repolarization/hyperpolarization when activated^15^.

The potential role of SACs in arrhythmogenesis is an active area of research^16^. Pharmacologic agents that modulate the gating behavior of SACs^17^ are a potentially powerful tool in this research and are therefore of great interest. Streptomycin is commonly used as an SAC blocker. However, while there is some inconsistency in the literature, it is typically reported to be effective in blocking SACs in isolated cells, but not in intact tissue^6,18–20^. Gadolinium (Gd^3+^) is another commonly used SAC blocker; it has been reported to modulate stretch-induced arrhythmias in intact myocardium, but again, the literature is inconsistent^21–23^. The purpose of the present study is to evaluate the ability of gadolinium to inhibit mechanically induced electrical activation in the specific case of whole, isolated large animal hearts subjected to localized stretch. To be an experimentally useful tool, gadolinium should have a substantial SAC-blocking effect. We did not evaluate a third agent, GsMtx-4, which is reported to be a specific SAC blocker^24^, but is not currently practical for this application because of the large quantity that would be required.

## Methods

### Heart Preparation

All animal protocols were approved by the University of Alabama at Birmingham Institutional Animal Care and Use Committee (APN 09818) and were in accordance with the Guide for the Care and Use of Laboratory Animals. Hearts were excised from animals in a surgical plane of anesthesia. Twelve hearts from domestic farm pigs of either sex, weighing 25–35 kg were studied. Anesthesia, heart excision, and perfusion were similar to our previous publication^25^. The hearts were retrogradely perfused in a Langendorff apparatus with warm (37 ± 1 °C), oxygenated perfusate at 100–200 mL/min. Hearts were suspended in air by the aortic cannula. A piece of concave-shaped polypropylene foam was placed against the posterior side of the heart for support during mechanical stimulation of the anterior left ventricular (LV) epicardium. Electrical activation of the heart was monitored and recorded using a silver wire unipolar electrode hooked into the subepicardium of the lateral LV wall. The reference electrode was immersed in perfusate in the aortic cannula.

Gd^3+^ strongly binds with anions such as bicarbonate and phosphate^26^ and quickly precipitates when added to commonly used perfusate solutions (such as Krebs-Henseleit or Tyrodes) that use bicarbonate as the buffer. To prevent this, we used HEPES-buffered perfusate in this study. The perfusate contained (mmol/L): NaCl 140, KCl 3, CaCl_2_ 2.6, MgCl_2_ 0.6, HEPES 10, Glucose 12. The perfusate was oxygenated with 100% O_2_ and the pH was adjusted to 7.4 ± 0.1 with NaOH. In six hearts, 3 L of perfusate was used. The coronary effluent was filtered with a 20 μm filter and recirculated. Gadolinium(III) chloride hexahydrate (Sigma-Aldrich) was dissolved in perfusate solution on the day of experiment. This solution was added to the perfusate in a serial manner with final Gd^3+^ concentrations of 0, 25, 50 and 100 μmol/L. Gd^3+^ has been reported to block SACs at concentrations of 1 to 50 μmol/L^7,22,23,27^. In the six remaining hearts, the coronary effluent was not recirculated to avoid possible precipitation of Gd^3+^ caused by metabolic wastes that the hearts produced. In those experiments, perfusate with different Gd^3+^ concentration was made and stored in four individual reservoirs. During the experiment, Gd^3+^ concentration was changed by switching the reservoir connected to the perfusion system. As before, concentrations of 0, 25, 50, and 100 μmol/L were tested. For analysis, 0 μmol/L data were used as the baseline group.

In all hearts, 2,3-Butanedione monoxime (BDM) (Sigma-Aldrich) was added to the perfusate (20 mmol/L) to suppress mechanical contraction while preserving electrical propagation. BDM also suppresses spontaneous electrical excitation of isolated hearts^28^. Thus, in this preparation, all cardiac wall stretch was induced experimentally and all electrical activations were either electrically paced or mechanically induced.

Once warm, hearts were defibrillated and electrically paced (twice diastolic threshold current) at 60 bpm by two silver wire electrodes hooked into the subepicardium at the LV apex. Whenever Gd^3+^ concentration was changed, 10 minutes of perfusion was allowed for equilibration.

### Mechanical Stimulation

**Figure 1:**
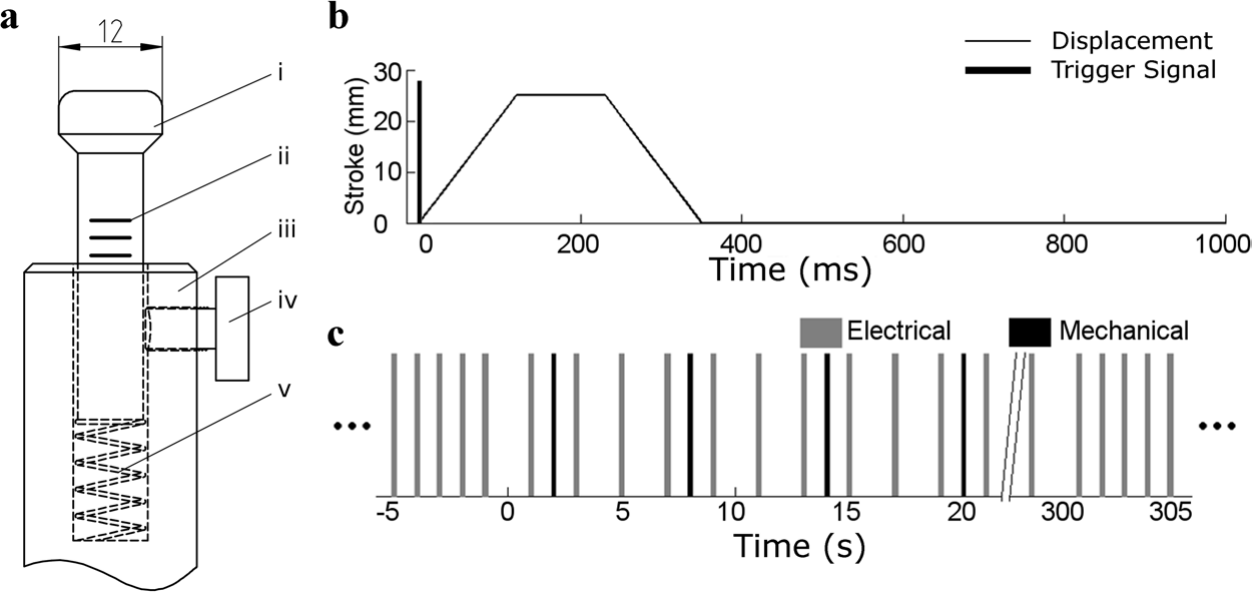
Mechanical stimulation protocol. (**a**) Dimensions of mechanical stimulation device. *i*, impact head (dimensions in mm). *ii*, markers for spring compression. *iii*, piston body (partial). *iv*, locking screw. *v*, spring. (**b**) Example of computer-controlled piston motion for delivering a mechanical stimulus. Bold bar indicates the timing of trigger signal for mechanical stimulation. Fine lines indicate piston movement. (**c**) Experimental protocol for delivering mechanical stimuli and electrical pacing. Gray bars indicate electrical pacing pulses and black bars indicate mechanical stimuli.

We elicited mechanically induced beats with a custom-built, computer-controlled piston that contacted the anterior LV epicardium midway between apex and base. The impact head at the end of the piston was approximately hemispherical with 6 mm radius (Fig. 1(a)). The mechanical stimulus strength was adjusted by altering the piston’s stroke length (with longer strokes inducing greater tissue deformation). Before each stimulus, the impact head was placed in an initial position that provided gentle contact with the epicardial surface. To provide for consistent initial contact force, the impact head was mounted on a spring. Every few stimuli, the locking screw was loosened, and if necessary, the piston’s initial position was adjusted to compress the spring to a consistent length. The locking screw was then tightened before stimulus delivery. The mechanical stimulation threshold strength (i.e. the threshold stroke length) under baseline conditions (0 μmol/L Gd^3+^) was found by gradually increasing the piston’s stroke. Stimulation success was noted when electrical activation was observed in the unipolar electrogram recorded from the lateral LV (5–10 cm from the mechanical stimulation site). Because the expected effect of Gd^3+^ was to reduce the likelihood of stimulation success, the stimulation stroke was adjusted until the majority of (≥70%), but not all, stimulation attempts at baseline were successful. Once the near-threshold stimulation strength was found for a heart, stimulus stroke was unchanged for the remainder of the experiment. The threshold stroke length varied from heart-to-heart, but was in the range 15–25 mm.

### Experimental Protocol

For each Gd^3+^ concentration level, a stimulation protocol containing ~50 (51 ± 8) mechanical stimuli (Fig. 1(b)) was performed. During the protocol, electrical pacing rate was reduced to 30 bpm and mechanical stimuli were delivered 1 s after every third or more electrical pacing pulse (Fig. 1(c)). The ~1 s delay between electrical and mechanical stimuli allowed myocardium to recover electrically before mechanical stimulus delivery. The unipolar electrogram and the trigger signals for electrical and mechanical stimulation were continuously recorded during the protocols. Electrical pacing resumed at 60 bpm between stimulation protocols.

### Data Availability

Data listing the outcome of each individual mechanical stimulus are listed in the supplementary data. Raw recordings are available from the corresponding author upon request.

## Results

**Figure 2:**
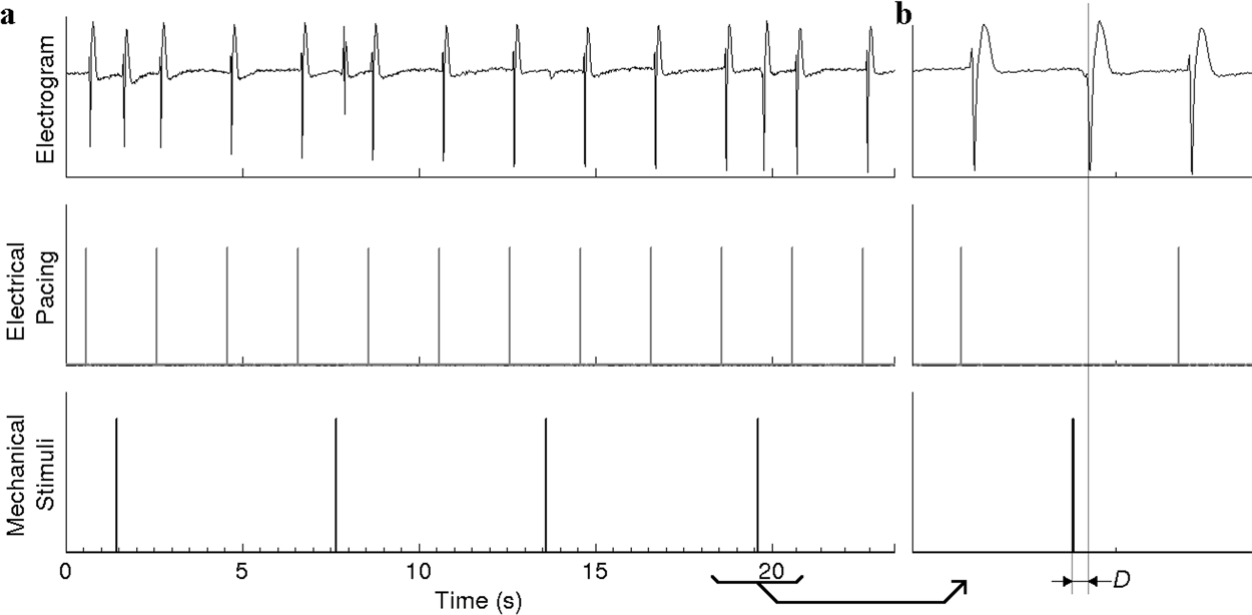
Example unipolar electrogram and electrical/mechanical stimulation trigger signals from a stimulation protocol. (**a**) The 1^st^, 2^nd^ and 4^th^ mechanical stimuli successfully elicited electrical activation; the 3^rd^ mechanical stimulus failed. (**b**) A localized view of the signals near 4^th^ mechanical stimulus in (**a**) shows the delay (*D*) between mechanical stimulation onset and electrical activation.

The successful and unsuccessful mechanical stimuli were identified from recordings of the left ventricular electrogram and the stimulus triggers. An example is shown in Fig. 2. The success rate for a mechanical stimulation protocol was defined as *R* = (*number of mechanically-induced electrical activations*)/(*number of mechanical stimuli*). We also measured the delay between the onset of mechanical stimulation and cardiac electrical activation (which we defined as the time of the steepest downslope of the unipolar electrogram signal^29^) for every successful mechanical stimulus (e.g., the interval *D* in Fig. 2(b)). Effective suppression of cardiac mechanosensitivity by SAC blockade should reduce *R* from its baseline value in a concentration-dependent manner and also increase the stimulation-activation delay *D* due to the decreased depolarizing current^30^. For analysis, we averaged *D* over the successful stimuli in each stimulus protocol. The results of all individual stimuli are shown in the supplementary data.

We used linear mixed models (SPSS MIXED procedure) to evaluate the effects of concentration. Gd^3+^ concentration was a repeated measure. Because mechanical stimuli were held constant for each trial in an animal and the status of the heart was approximately constant throughout an experiment, we considered as plausible the assumption that variance and covariance of residuals among levels of concentration were equal. We therefore used the “compound symmetry” covariance structure when fitting models^31^.

**Figure 3:**
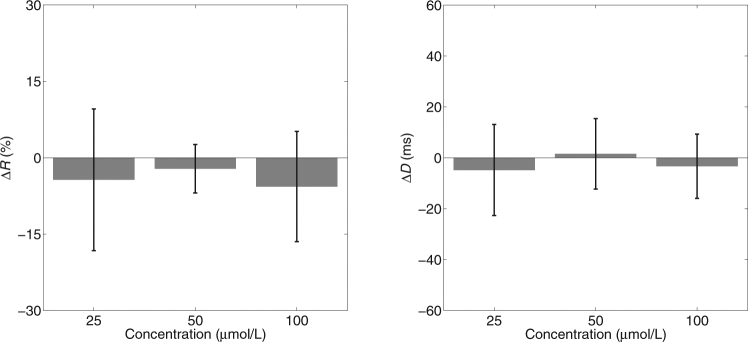
Effects of Gd^3+^ on mechanical stimulation success rate (*R*) and stimulation-activation delay (*D*) with recirculated perfusate. For each concentration level, *R* and *D* in the baseline condition (i.e. concentration = 0) were subtracted from *R* and *D* and the change averaged over animals is displayed. Error bars are standard deviations.

The effect of Gd^3+^ for the hearts in which perfusate was recirculated is shown in Fig. 3. To be experimentally useful, SAC blockade should have a substantial effect. However, the linear mixed model analysis did not indicate any significant differences in *R* or *D* among the 4 levels of Gd^3+^ concentration (*p* = 0.614, *p* = 0.821, respectively).

**Figure 4:**
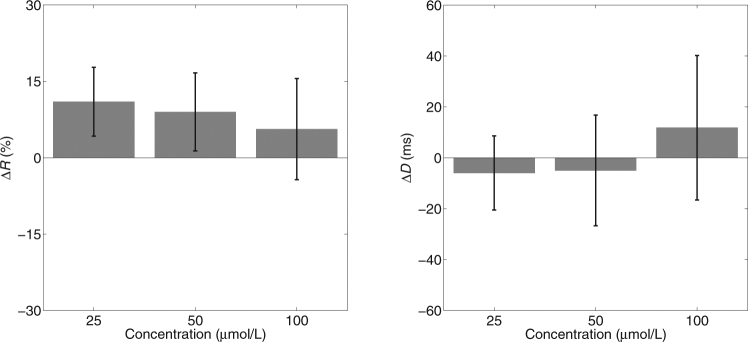
Effects of Gd^3+^ on mechanical stimulation success rate (*R*) and stimulation-activation delay (*D*) with non-recirculated perfusate. As in Fig. 3, change from baseline averaged over animals is displayed. Error bars are standard deviations.

We observed that when Gd^3+^ was added to fresh perfusate with high concentration (7.5 mmol/L), it remained dissolved. However, when a similar concentration was added to perfusate that had been recirculated through a heart during an experiment, a visible precipitate formed, possibly through interaction with metabolic wastes that had washed into the solution. To avoid this potential for loss of Gd^3+^, we also tested the effect of Gd^3+^ in six hearts *without* perfusate recirculation. These data are shown in Fig. 4. The 100 μmol/L data from one heart was lost due to a technical issue. Mixed model analysis in this case indicated that there *was* a significant difference in *R* among the levels of Gd^3+^ concentration (*p* = 0.006). Examination of the pairwise comparisons between baseline (0 μmol/L Gd^3+^) and the other three levels showed significant differences with 25 and 50 μmol/L Gd^3+^ but not with 100 μmol/L Gd^3+^ (*p* = 0.003, *p* = 0.015, *p* = 0.227, respectively with Sidak adjustment for multiple comparison). However, in all 6 animals, *R* was increased, rather than decreased, by 25 and 50 μmol/L Gd^3+^, which is the opposite of the hypothesized effect. Mixed model analysis did not indicate a significant effect of Gd^3+^ concentration on *D* (*p* = 0.286).

To investigate possible loss of mechanical stimulation efficacy with repeated delivery (“mechanical rundown”)^32,33^, we compared the stimulation-activation delay *D* between the first and second mechanical stimuli in each stimulation protocol and also between the two stimuli immediately preceding each failed mechanical stimulus. *D* was significantly shorter for the first stimulus compared to the second (154 ± 43 vs. 202 ± 48 ms, mean ± stdev, *p* < 0.001, paired *t*-test, n = 46 pairs), but there was not a significant difference between *D* of the two stimuli preceding failure (209 ± 54 vs. 211 ± 56 ms, mean ± stdev, *p* = 0.62, paired *t*-test, n = 214 pairs).

## Discussion

The major finding of the present study is that gadolinium (Gd^3+^) does not reliably suppress electrical excitation due to focal mechanical stimulation in whole isolated swine hearts.

Gadolinium (Gd^3+^) is frequently used as a SAC blocker. It has been reported to block SACs at concentrations of 1 to 50 μmol/L^7,22,23,27^. Other lanthanides (La^3+^ and Lu^3+^) also show SAC-blocking effects, but higher concentrations are required^27^. The mechanism of action of Gd^3+^ is not yet clear and multiple mechanisms and sites of action may be involved^34^. Gd^3+^ is not a specific SAC blocker. Within the effective concentration range, it also blocks many other currents in cardiac cells, including calcium, sodium and potassium currents^17^. However, some studies have reported an inability of Gd^3+^ to block SACs. Zhang *et al*. reported that Gd^3+^ (100 μmol/L) can block background non-selective cation current but failed to block stretch-induced non-selective cation current in rat atrial myocytes. In the same study, they also demonstrated that action potentials can still be elicited by mechanical stretch after Gd^3+^ treatment^35^. Similarly, Kim reported that SAC current in rat atrial cells cannot be blocked by 100 μmol/L Gd^3+ ^^36^. In the present study, Gd^3+^ failed to suppress mechanically induced electrical excitation in whole isolated hearts (Figs 3 and 4), which is consistent with those findings. Furthermore, Gd^3+^ did not significantly prolong the delay between the onset of mechanical stimulation and electrical activation. Stacy *et al*. reported that the delay between mechanical stimulation and electrical activation as well as the amplitude of mechanically induced depolarization depended on the strength of the stimulation^37^. Thus, if Gd^3+^ successfully attenuated depolarizing current resulting from mechanical stimulation, it would be expected that *D* would increase in a concentration dependent manner, even if the attenuation was insufficient to prevent electrical activation.

The efficacy of Gd^3+^ in some studies but not others may suggest that there are sources of mechanosensitivity other than channels susceptible to block by Gd^3+^. The lack of activity of Gd^3+^ in our study could also have been due to failed transport of the agent to its site of action. This broad mechanism was suggested by Cooper and Kohl to explain similar inconsistency of streptomycin in suppressing mechanosensitivity^6^. Gd^3+^ is known to strongly bind with anions that commonly exist in physiological solutions (e.g. carbonate, phosphate, EGTA, etc.). Such binding effectively removes free Gd^3+^ from the solution^26^. This is why we used a phosphate-free HEPES-based perfusate solution, which lacks these anions. However, in a recirculating perfusion system, the possibility remains for Gd^3+^ to bind with metabolites and other substances washed into the perfusate. Indeed, we observed that when Gd^3+^ was added to *used* HEPES-based perfusate, visible precipitate formed. In a typical recirculating perfusion system, precipitate would be quickly filtered from the working solution. We therefore additionally tested Gd^3+^ using non-recirculating perfusate. These experiments also found a lack of effect, suggesting that anion-binding was not responsible for reducing the efficacy of Gd^3+^. It has been proposed that Gd-anion complexes rather than free Gd^3+^ are responsible for blocking SACs and that this could partly explain the reported positive effects of gadolinium in the presence of suspect anions^17,26^. If this is the case, it is possible that SAC block by gadolinium occurs under conditions in which Gd-anion complexes form, but are not rapidly removed from solution by precipitation and filtration.

Use-dependent decrease of mechanical stimulation efficacy (also called “mechanical rundown”) has been reported^32,33^ and is a potential cause for failed mechanical stimulation in repetitive protocols such as ours. The sustainability of successful mechanical stimulation depends on the presence of background electrical pacing and decreases as pacing rate increases^32^. In our study, mechanical stimuli were interspersed with electrical stimuli at a ratio ≥3:1 (electrical:mechanical) and the hearts were paced at a rate lower than normal pig sinus rhythm^38^. This pacing condition facilitates the sustainability of successful mechanical stimulation^32^. In our experiments, we observed mechanical rundown at the beginning of each stimulation protocol (increased *D* for the second stimulus compared to the first), but the phenomenon was not observed as mechanical stimulation approached failure (no difference between *D* of the two stimuli preceding a failed mechanical stimulus). This is consistent with Quinn and Kohl’s observation that rundown was pronounced after the first mechanical stimulation but less prominent thereafter^32^. It is therefore unlikely that mechanical rundown played an important role in our results.

Myocardial edema is common in isolated heart preparations as a consequence of interstitial fluid accumulation and cell swelling^39^. It is known that cell swelling affects several ion channels, including volume-sensitive channels (e.g. Cl-selective volume-sensitive channels)^40^ as well as SACs^36^ in cardiac cells. However, in this study, the most important effect of edema was likely on the heart’s mechanical properties (i.e., stiffness). Changing stiffness alters tissue deformation patterns resulting from the mechanical stimulus and could modulate the electrical response to stretch. Because swelling is most rapid immediately after starting perfusion, to minimize this variability, after isolating the hearts and initiating perfusion, we allowed the hearts to stabilize for ~40 min before proceeding to the remaining experimental protocols.

In our experiments, there was no visible tissue damage (e.g. tissue tearing, rupture) from mechanical stimulation; however it is possible that non-visible damage occurred. Cooper *et al*. reported that creatine kinase elevation, an indicator for muscle tissue damage, was observed when focal mechanical impact exceeded a certain energy level^41^. However, the energy level required for triggering electrical activation generally does not cause such tissue damage^20,32^. In the present study, the mechanical stimulation strength was maintained at a level just sufficient to elicit electrical activation.

We used BDM to prevent stretch from muscle contraction and to suppress spontaneous electrical activation. BDM is known to affect cardiac electrophysiology, including calcium and potassium currents, action potential duration, and ventricular fibrillation patterns^25,42^. Although no evidence has been reported suggesting that BDM affects the gating behavior of SACs, it is possible that it interfered with SAC blockade.

Many studies have shown that cardiac mechanosensitivity can be arrhythmogenic, e.g., ventricular fibrillation initiated by non-penetrating impact on the chest^3^ or ectopic beats elicited by mechanical stretch of the heart wall^4^. It has been suggested that abnormal mechanical stretch at the interface of normally contracting and ischemic tissue following acute regional ischemia may play a role in arrhythmogenesis^43,44^. Pharmacologic agents to suppress mechanosensitivity are therefore of great interest, both as experimental tools to probe the mechanisms of these phenomena, and as possible therapeutic agents to prevent arrhythmia. However, studies evaluating the ability of SAC blocking agents to prevent arrhythmia have yielded inconsistent results. Bode *et al*. showed that GsMtx-4, a more specific SAC blocker than that used in the present study, effectively inhibited atrial fibrillation following mechanical stretch^24^. Similar antiarrhythmic effects have also been reported after streptomycin and Gd^3+^ treatment^18,22,23^. On the other hand, antiarrhythmic effects of these SAC blockers were not observed in other studies^19–21,45^. In the present study, we used a large animal heart preparation in which confounding sources of wall stretch and electrical activation were absent. We mechanically stimulated the heart with controlled, repeatable, focal stimuli. Our results indicate that Gd^3+^ is not effective in eliminating ectopic beats due to local stretch of the ventricular wall and should not be relied upon in this role experimentally.

## Electronic supplementary material

Dataset 1
